# Commissioning experience and quality assurance of helical tomotherapy machines

**DOI:** 10.4103/0971-6203.56078

**Published:** 2009

**Authors:** Amarjit Sen, Matthew K. West

**Affiliations:** Department of Radiation Oncology, Cancer Treatment Centers of America, Tulsa, OK 74133, USA

**Keywords:** Beam commissioning, helical tomotherapy, image-guided IMRT, quality assurance

## Abstract

A helical tomotherapy machine combines a straight 6 MV linear accelerator mounted on a ring gantry with CT technology for image-guided intensity-modulated radiation therapy (IMRT) treatment. A fan beam created by the collimator and jaws produces a maximum of 40 × 5 cm^2^ field size at the isocenter. The gantry and hence the fan beam rotates at a constant speed while the couch moves linearly into the gantry bore, thus producing a helical delivery. The beam is modulated by a 64-leaf binary multileaf collimator (MLC), which enables IMRT treatment. The linac can be operated at a lower voltage (3.5 MV) and dose rate to produce megavoltage CT images, which are used for image-guided patient setup. We have installed two such units since 2004 and treated more than 2000 patients. The machine comes “precommissioned” from the manufacturer, and the beam characteristics and IMRT plans on phantom are measured and compared with manufacturer's data after acceptance tests are performed on site. Our experience with commissioning the machines and periodic quality assurance with tolerance limits for optimal performance are described.

## Introduction

**Figure 1:**
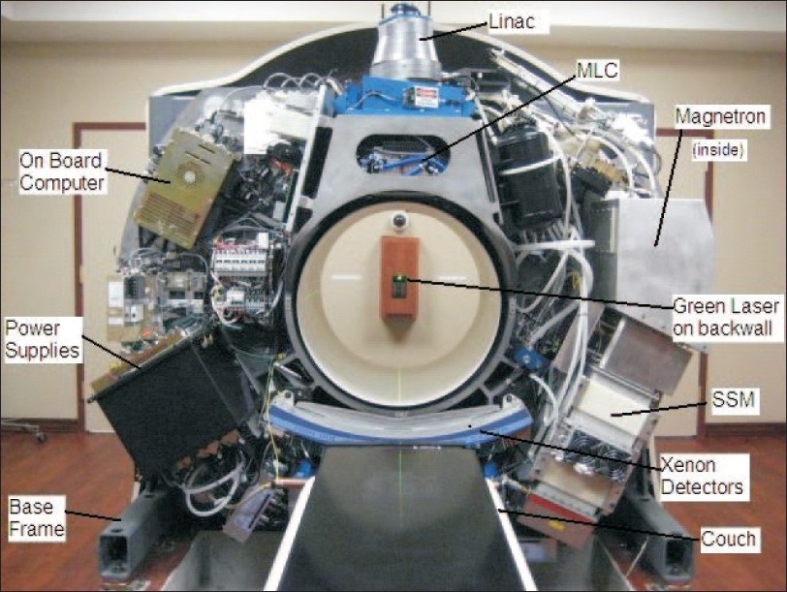
Tomotherapy HI ART II machine

The concept of a helical tomotherapy machine for radiation therapy was originally proposed by Mackie *et al*.[1] in 1993, and the first commercial machine was installed 10 years later. It combines the conventional linac technology with CT technology for image-guided intensity-modulated radiation therapy (IMRT) treatment. An in-line straight 6-megavoltage (MV) linac mounted on a ring gantry and powered by a magnetron rotates continuously while the horizontal couch carrying the patient moves linearly in to the gantry bore [Figure 1], thus producing a helical beam delivery. The beam is fan-shaped, produced by a set of collimating jaws with maximum transverse length of 40 cm and a selectable width up to 5 cm in the longitudinal direction at the isocenter. There is no flattening filter and the beam is cone shaped in the transverse direction. This produces an increased dose rate of about 850 cGy/min at the isocenter. The fan beam passes through a 64-leaf binary multileaf collimator (MLC) driven pneumatically, thus producing 64 beamlets for beam modulation. The fan beam is intercepted by a set of 640 xenon detectors mounted on the ring gantry opposite to the x-ray source similar to those used in a CT scanner. These detectors are used for reconstruction of the MVCT images, dose delivery verification, performance checks and quality assurance of the machine. A 13-cm-thick lead beam stopper is mounted behind the xenon detectors on the ring gantry for beam attenuation. Unlike a conventional radiotherapy linac there is no light field for patient setup. Instead, the patient is set up with the help of two fixed green lasers and five red movable lasers intersecting at a virtual isocenter on the axis of gantry rotation, 70 cm away from the real isocenter. Therefore, the alignment of the lasers is critical to treatment planning and delivery. The isocenter is 85 cm from the source (SAD), which is also the bore diameter. A shorter SAD also helps increase the dose rate. A comparison between the tomotherapy machine and a conventional linac is given in Table 1.

**Table 1:** Comparison of tomotherapy machine and a conventional linac

*Tomotherapy Hi ART*	*Conventional linac*
No flattening filter	Has flattening filter
Cone beam	Flat beam
Beam up to 5 × 40 cm^2^	Beam up to 40 × 40 cm^2^
85 cm SAD	100 cm SAD
No light field	Has light field
Fixed and movable lasers	Fixed lasers
Xe detector array	No such detectors
MVCT mode for imaging from	kVCT mode for imaging with the
same source	additional source
Pneumatically driven MLC leaves	Motorized MLC leaves
Helical treatment	Fixed gantry or arc therapy
Inverse planning only	Forward and inverse planning

The machine operates in two modes: (1) imaging mode and (2) treatment mode. In the imaging mode, the linac is operated at a lower accelerating potential (3.5 MV) and a substantially lower dose rate to produce the MVCT images, which are used for coregistration with kVCT images on a daily basis for accurate positioning of patients. The treatment mode uses the 6-MV beam and higher dose rate for helical IMRT delivery.

At Cancer Treatment Centers of America, Tulsa, we have installed two Tomotherapy Hi ART II machines since July 2004. We have treated more than 2000 patients as of July 2008 at sites such as prostate, brain, lung, breast, spine, liver, esophagus, head and neck, etc. We have also performed special procedures such as stereotactic radiosurgery (SRS), stereotactic body radiotherapy (SBRT) and craniospinal treatments.

## Materials and Method

Before using a machine for radiotherapy treatment, following checks are performed in general: (1) acceptance testing, (2) dosimetric commissioning, (3) plan verification, (4) quality assurance procedures and (5) radiation and other safety. After installation of a machine, acceptance testing procedures are performed usually with guidelines from the manufacturer to verify that the machine performs within the specification of the manufacturer. After that in dosimetric commissioning, beam data are obtained by scanning measurements, which are used in modeling and treatment plan delivery. Several treatment plans are generated on suitable phantoms and delivered on the phantoms for point dose and dose distribution measurements. When satisfactory criteria for treatment plan generation and delivery are met, the machine may be used for actual patient treatment. A set of periodic quality assurance checks are established according to professional and regulatory guidelines for proper performance checks of the machine. Of course, most radiotherapy machines for treatment are operated under strict guidelines from regulatory authorities for radiation safety and other safety for patients and members of the public.

For dosimetric commissioning of a conventional linac, beam characteristics of the machine are measured after checking the optimal performance of the machine. This involves measurement of scan and non-scan data. For scan data, central axis percentage depth-dose scans and profile scans at several depths for open and wedged fields are measured with a suitable water scanning system. The non-scan data such as output factors, wedge factors, tray factors, transmission factors, etc., are also measured. Absolute dose calibration is then performed for reference conditions, using professional protocols (AAPM TG-51 or IAEA TRS-398). Using beam spectra and measured beam data, a beam model is generated by an iterative process. Treatment plans are generated on a phantom and delivered for verification of absolute dose at selected points and dose distribution on suitable planes.

Tomotherapy machine comes precommissioned to the site from the manufacturer. Upon installation, the site physicist performs a series of acceptance tests to verify that the machine functions within the specifications. The beam scan data are then measured with a water scanning system to verify that they match with those measured by the manufacturer at their bunker. If there are major differences, the machine is “tweaked” in order to reproduce the bunker data. At this time, IMRT plans are generated, measured on phantoms and compared for acceptable delivery. A physicist from the Tomotherapy Inc., assists in these measurements.

The beam modeling of Tomotherapy machine involves four steps: (1) static beam profiles, (2) helical data, (3) MLC data and (4) IMRT plan verification.

Static profiles: A water scanning system (Standard Imaging Corporation) is appropriately set up in the gantry bore and normalized profile scans are obtained in three orthogonal directions to generate percentage depth dose, cone and penumbra profiles. PDDs are used to quantify the spectral content (energy distribution). Cone and penumbra profiles are used to shape the beam model in the transverse and longitudinal directions. Iterative process is used till the calculated and measured profiles match.

Helical data: Helical data allow a unique self-consistent method to commission a tomotherapy machine. Ten rotational helical open beams are delivered for each beam width, forcing the model to produce the same dose as simulated. For self-consistency, various rotational (1, 2, 3, …9 rotations) helical deliveries are tried for calculating and measuring absolute fluence levels. If there are errors in the model, the superposition of the beams in the longitudinal direction will magnify the error. Now the calculated and measured data match in absolute value and shape.

MLC data: Up to this point, open beams are used for modeling. MLC leaves are opened and closed pneumatically and take finite amount of time (< 20 ms) to fully open or close. Also, additional fluence is included when two neighboring leaves are open simultaneously as opposed to separately. This information is measured as leaf-fluence output factors and is stored in the database for planning calculation.

**Figure 2:**
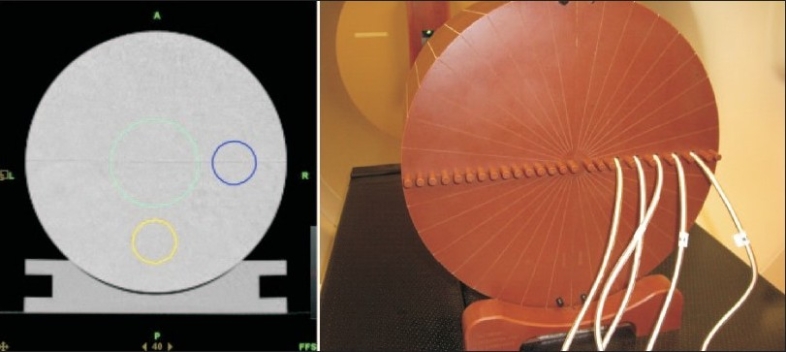
Cylindrical on-axis and off-axis targets and cheese phantom setup for measurement. The CT image of the cheese phantom (left) shows an on-axis and two off-axis target structures. The cheese phantom has multiple holes in which A1SL ion chambers can be inserted for simultaneous dose measurement at multiple points as shown on the right

IMRT plan verification: IMRT plans are generated for cylindrical targets on a “cheese” phantom for “on-axis” and “off-axis” locations for each beam [Figure 2]. Point dose is measured at several points at high-dose and low-gradient, and low-dose high-gradient locations using multiple ion chambers and multichannel tomoelectrometer. Dose distributions are measured by putting an extended dose range film in a selected plane in the cheese phantom, and dose profiles are generated in transverse and longitudinal directions for comparison. The model is adjusted till the calculated and measured data match and IMRT plan measurements are repeated as needed.

## Results

The acceptance tests are performed after all the alignment tests and dosimetric consistencies are checked. The purpose of these tests is to verify proper functioning of the system per manufacturer's specification, MVCT imaging and safe and accurate treatment delivery. A brief description of these tests along with the tolerance criteria is given in Table 2.

**Table 2:** Acceptance tests of a tomotherapy Hi-ART machine

*Items checked*	*Acceptance method*	*Acceptance check*
System start up	- Upon power up the red lasers turn on and move to home position	Functional
	- Operator station (OS) computer turns on and operator logs in	
	- System initializes, couch and laser positions are displayed on position control panel	
System shut down	- Operator station computer turned off, key turned off on the machine and switched off. Red lasers turn off and the power distribution unit is turned off	Functional
Manual couch control	- Couch moves up or down and in or out only when the enable button underneath is pressed	Functional
DICOM import	- Import of DICOM images (kVCT) and structures to the system and display at the operator and planning station (PS)	Images can be viewed at OS and PS
Safety interlock	- Mode switch: Imaging MVCT can not be formed when the mode switch is at treat	Functional
	- Visual and audible indicators working	
	- Door interlock working – checked during MVCT imaging procedure	
	- Stop button on position control panel (PCP) working	
	- Each emergency stop inside and outside the treatment room and on status control shuts down power to the gantry	
Laser alignment	Verify home positions for movable red lasers where they overlap the fixed overhead green lasers within ±1 mm	Verified
	Create a MVCT scan procedure for the cylindrical cheese phantom with the dowel at 5 mm position out. Red lasers move from home position to 2, 8 and 4 cm in the X, Y and −Z directions, respectively.	
	Dose during MVCT scan is less than 4 cGy	
	MVCT images are reconstructed and viewable from Reconstruction tab.	
	Lateral and vertical shift for the reference “cross” on the MVCT image is ±1 mm and 5±1 mm, respectively.	
Treatment delivery test	Treatment plans for “on axis” and “off axis” targets on a cheese phantom are delivered. The measured dose at each point is within ± 3% at ± 3 mm of the calculated point.	Verified
Interrupted treatment completion test	Two films are exposed sequentially with uninterrupted and interrupted but completed procedures. Visual inspection of the films shows no discontinuity and point dose difference is within 3%.	Verified

**Figure 3 A-C:**
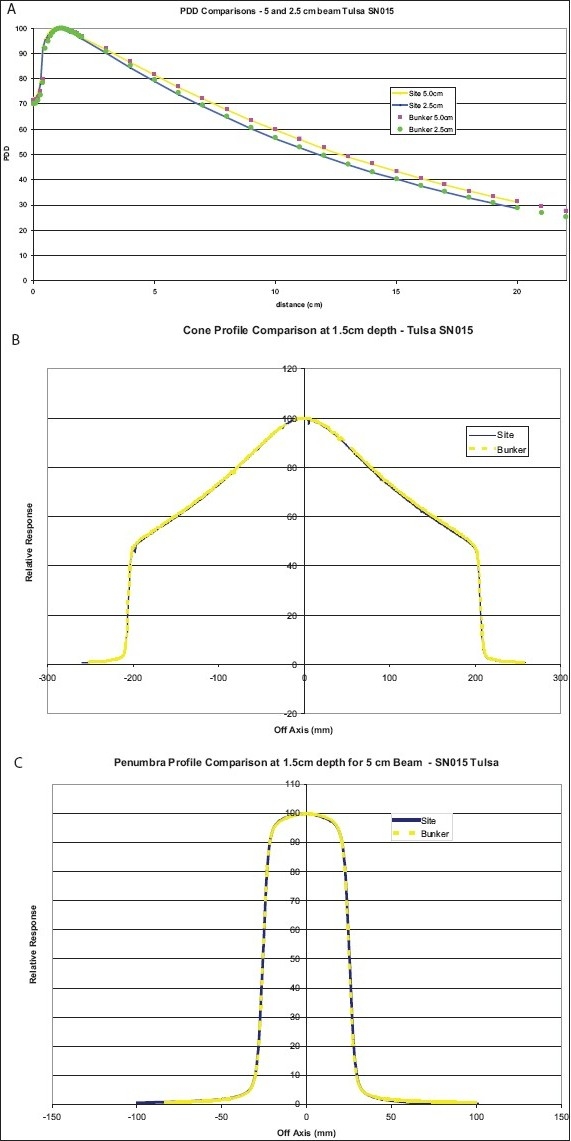
Match of PDD, cone and penumbra profiles measured on site and at manufacturer's bunker

**Figure 4 (A) and (B):**
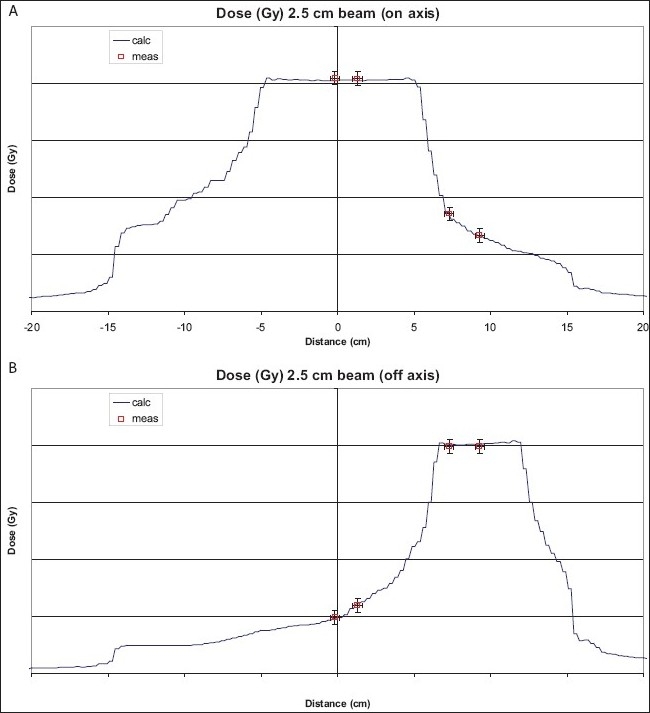
Comparison of point dose measurements at on-axis and off-axis targets in a cheese phantom

Upon completion of installation and acceptance testing, the static beam profile scans are performed and compared with those obtained in the bunker by the manufacturer. Examples of the agreement of the PDD, cone profile and penumbra profile are shown in Figure 3 A–C. After matching of the helical data, the IMRT plans for on-axis and off-axis targets are delivered for each of the three beams (i.e., 5-, 2.5- and 1-cm beam) in a cheese phantom. Point doses measured in the high-dose low-gradient, and low-dose high-gradient regions are shown in Figure 4 (A) and (B) for on-axis and off-axis targets, respectively. The calculated beam profiles through the target are also shown in the figures. The agreement of the point doses are within 1%. An extended dose range film is also placed in a coronal plane between the two halves of the cheese phantom when the treatment is delivered for the on-axis and off-axis targets. The film is processed and analyzed with a film analysis software, and compared with the calculated profiles from the plan, showing a good agreement.

The MVCT images are checked for minimum dose, resolution and artifacts. The beam characteristics and benchmarking alignment tests have been reported by Jeraj *et al*.[2] and Balog *et al*.,[3] respectively. The stability of the beam output is an issue, which we have been monitoring by sampling the beam output at 100-ms intervals over an extended period of time (West *et al*.[4]).

## Quality Assurance

Suggestions for periodic comprehensive quality assurance (QA) of radiotherapy machines have been made by AAPM Task Group 40 report. Fenwick *et al*.[5] have published a set of quality assurance tests exclusively for a tomotherapy machine. We have developed a set of quality assurance tests performed at regular intervals for monitoring optimal performance of our machines.

The quality assurance of the machine is performed on a daily, weekly, monthly and annual basis which is listed in Table 3 along with the acceptable tolerance limits. In addition, the service engineer from Tomotherapy performs periodic preventive maintenance tasks on a regular basis.

**Table 3:** Quality assurance of a tomotherapy Hi-ART machine

*QA*	*Item*	*Description*	*Performed by*
Daily	Temperature	Gantry temperature control system reads 40°C after machine is turned on.	Radiation
QA	Warm up procedure	Beam runs at lower output for 10 min and warms up the components to stable temperature.	Therapist
	Air Scan	With the couch out of the beam, the machine is run in the imaging mode, which corrects for the detector response.	
	Laser check	Green and red lasers fall on top of the marks on walls and floor created during commissioning.	
		Green and red lasers coincide at the virtual isocenter in the home position.	
	Output check	With a nine-chamber electrometer (Victoreen 7200) output is measured for a 5 × 20 cm^2^ open field on central axis and two off-axis points on each shoulder of the cone profile, to within ±3%.	
	Door Interlock	Machine cancels procedure when the entrance door is opened.	
Weekly QA	Output check	With an A1SL ion chamber and electrometer the output is measured for a 5 × 20 cm^2^ open field at 1.5 cm depth in solid water in SAD setup (tolerance ±3%).	Physicist
	Energy check	A 4-cm solid water slab is placed on the previous setup and the ratio of the two readings gives a measure of the beam energy at 5.5 cm depth (tolerance ±2%).	
	Laser check	With the cheese phantom, the transverse laser (X-Z plane) localization procedure is performed in which MVCT scans are done. During setup, the red lasers move by 2, 8 and 4 cm in the X, Y and −Z directions, respectively. From the scans and registration of images, the X, Y and Z shifts are within 0 ± 1, 0 ± 1 and 5 ± 1 mm, respectively.	
Monthly	Output check	Same as in weekly QA	Physicist
QA	Energy check	Same as in weekly QA	
	Laser check	Same as in weekly QA	
	Virtual isocenter check	With a ready pack film set up horizontally at the virtual isocenter and the green laser line marked on the film, the procedure moves the film 70 cm into the bore and exposes for an open field. The center of the exposure is within ±0.5 mm of the laser line.	
	Penumbra profile check	With a ready pack film set up horizontally at isocenter at 1.5 cm depth and SSD of 85 cm in solid water an exposure is made for all open leaves for each beam. The penumbra profiles are measured to within ±1 mm of FWHM at commissioning.	
	IMRT plan check	IMRT plans are measured for cylindrical targets in a cheese phantom for on axis and off axis locations to within ±3% for the 1, 2.5 and 5 cm beam.	
	Output drift check	With an ion chamber and Tomoelectrometer, an open beam at fixed gantry is sampled for every 100 ms for 10 min. The converted dose rate should be within ±2% of the average value.	
	Couch motion	A 40 × 1 cm^2^ open field is exposed on a film for the gantry at 0° and the couch moving at a constant speed for 20 cm. The optical density scan in the Y-direction should be constant within ±2% for constant speed and output.	
	Rotational variation	A procedure to monitor the beam output and energy is carried out by measuring the signal on the monitor chamber of the machine and the xenon detectors with the help of service engineers (tolerance ±2%).	
	Safety interlock	As described in the acceptance tests.	
Annual QA	Alignment checks	Jaw shift (tolerance ± 0.3 mm), Y-axis misalignment (tolerance ± 0.5 mm), MLC center – Gantry isocenter alignment (tolerance ± 0.5 mm), Tongue and groove test (tolerance <2%) are checked.	Physicist
	Beam Characteristics	Water scanning system is set up to measure (1) PDD, (2) Cone profiles and (3) Penumbra profiles at several depths for all the three beam widths and compared with commissioning data.	
	Displayed dose rate	Absolute dose output is measured for a set time and dose rate is calculated. This is set as the displayed dose rate at the operator station.	
	MVCT images	Tomoimage reconstruction is examined for coarse, normal and fine settings for no artifacts and good contrast resolution. MVCT image dose should be < 4 cGy.	
	Output check	With an A1SL ion chamber in water medium, the output is measured for a 5 × 40 cm^2^ beam at 1.5 cm depth in SAD set up (tolerance ± 2%).	
	Energy Check	The ion chamber is moved to a depth of 10 cm in water and the readings taken in SAD set up. The ratio of the readings in the previous step and this is a measure of the energy check (tolerance ± 2%).	
	Laser check	Same as in monthly QA	
	Virtual isocenter	Same as in monthly QA	
	IMRT plans	Same as in monthly QA	
	Output drift	Measure over 1000s (drift tolerance within 2%)	
	Safety interlocks	Same as described in Acceptance test	
	Couch motion	Same as in monthly QA	

Daily QA: The daily QA is performed by a trained therapist in the morning before beginning any patient treatment. The machine is warmed up after manufacturer's warm-up procedure. The positions of the green and red lasers are then checked to be coincident with the marks created on the walls and floor at the time of acceptance testing. The red lasers are checked to match the green lasers in the home position in appropriate directions. Finally, accurate displacements of the red laser lines are checked using a known procedure. The machine output is measured with a 9-chamber Victoreen 7200 daily checker device for a field size of 20 × 5 cm^2^. This also enables to measure the constancy of the shoulders of the cone profile at two off-axis points on each side of the central axis. The daily QA checks are reviewed by a qualified physicist.

Weekly QA: The output of the machine is checked by a physicist with an A1SL ion chamber and solid water phantom for a 20 × 5 cm^2^ field size for irradiation time of 60 s. From this, the dose rate, in cGy/min, of the machine is checked quickly. The dose is also measured at a depth for checking the energy constancy. The lasers are checked after a transverse laser (x-z plane) localization procedure provided by the manufacturer.

Monthly QA: In addition to the steps in weekly QA, a few more checks are performed with films. The position of the virtual isocenter is performed by an overhead laser alignment procedure in which a film is set up horizontally at the isocenter plane in a solid water phantom at the virtual isocenter and allowed to move 70 cm into the bore in which an exposure is made for an open field. The green laser position on the film at virtual isocenter is marked on the film, and the alignment of the laser line with the edge of the exposed open field on the film is found to be less than 0.5 mm. Also, penumbra profile for an open field is measured at a depth for each beam and compared with commissioned values. Finally, the IMRT plans for on-axis and off-axis points for each beam width are measured with the cheese phantom.

Annual QA: Most of the alignment checks for the source, jaws and MLC, which were originally performed during acceptance test are performed at this time. The PDD and profiles are measured with the help of a water scanning system as part of verification of original beam data. The calibration of displayed monitor units for treatment and imaging mode are also checked. The contrast resolution of the tomoimage quality, image artifacts and dose delivered during MVCT are also checked. Finally, the safety interlocks are checked for compliance with regulations.

The weekly machine outputs versus time are plotted annually for each machine and are found to be within 2% of the average value. For the patient-specific delivery QA plans, the average point dose measurement is within 0.3% of the calculated values, with a spread between -5% and 5% at the extreme limits. When the delivery QA point dose measurement exceeds 5%, the measurement is repeated. The gamma analysis of the 2D-dose distribution from the film measurement is done with an acceptance limit of ±3% dose difference and ±3 mm distance to agreement.

On occasions when a major component such as the linac or the target is changed, a comprehensive set of checks performed during the commissioning time is repeated for bringing the machine back to original condition.

## Conclusion

We have performed acceptance tests and commissioning of two helical Tomotherapy Hi ART machines in the last four and half years and developed a comprehensive quality assurance program for optimal performance evaluation. The QA program has evolved over the years and different tests and their frequencies have been added as needed. Various acceptance tests and the QA checks have been designed and are presented here in tables with tolerance limits for the benefit of the readers. We have confidently treated over 2000 patients at various treatment sites and performed special procedures such as craniospinal treatment, stereotactic radiosurgery and stereotactic body radiotherapy of the lung.
